# Human Relaxin-2 (Serelaxin) Attenuates Oxidative Stress in Cardiac Muscle Cells Exposed In Vitro to Hypoxia–Reoxygenation. Evidence for the Involvement of Reduced Glutathione Up-Regulation

**DOI:** 10.3390/antiox9090774

**Published:** 2020-08-21

**Authors:** Silvia Nistri, Claudia Fiorillo, Matteo Becatti, Daniele Bani

**Affiliations:** 1 Department of Experimental & Clinical Medicine, Research Unit of Histology & Embryology, University of Florence, viale G. Pieraccini 6, 50139 Florence, Italy; silvia.nistri@unifi.it; 2 Department of, Experimental & Clinical Biomedical Sciences “Mario Serio”, Section of Biochemical Sciences, University of Florence, viale G.B. Morgagni 50, 50134 Florence, Italy; claudia.fiorillo@unifi.it

**Keywords:** relaxin, serelaxin, cardiac muscle cells, ischemia-reperfusion, hypoxia–reoxygenation, oxidative stress

## Abstract

Serelaxin (RLX) designates the pharmaceutical form of the human natural hormone relaxin-2 that has been shown to markedly reduce tissue and cell damage induced by hypoxia and reoxygenation (HR). The evidence that RLX exerts similar protective effects on different organs and cells at relatively low, nanomolar concentrations suggests that it specifically targets a common pathogenic mechanism of HR-induced damage, namely oxidative stress. In this study we offer experimental evidence that RLX (17 nmol L-1), added to the medium of HR-exposed H9c2 rat cardiac muscle cells, significantly reduces cell oxidative damage, mitochondrial dysfunction and apoptosis. These effects appear to rely on the up-regulation of the cellular availability of reduced glutathione (GSH), a ubiquitous endogenous antioxidant metabolite. Conversely, superoxide dismutase activity was not influenced by RLX, which, however, was not endowed with chemical antioxidant properties. Taken together, these findings verify the major pharmacological role of RLX in the protection against HR-induced oxidative stress, and shed first light on its mechanisms of action.

## 1. Introduction

Serelaxin (RLX; synonyms: recombinant human relaxin, rhRLX) designates the pharmaceutical form of the human natural hormone relaxin-2 (RLX-2; H2 RLX) suitable for clinical use. In recent years, robust evidence has been accumulating that RLX can markedly reduce tissue and cell damage induced by ischemia and reperfusion (IR) [1,2,3]. Such a protective effect has been consistently observed in diverse experimental models, spanning from organ IR in whole animals (heart, gut, kidney) [4,5,6,7,8] and in isolated and perfused organs (heart, lung, liver) [9,10,11,12] to specific cell types (cardiomyocytes, trophoblast) subjected to in vitro hypoxia (associated or not with nutrient starvation) and reoxygenation [13,14]. The fact that RLX exerts similar protective effects on different organs and cells at relatively low concentrations (in the nanomolar range) suggests that this molecule specifically targets a common pathogenic mechanism of the IR-induced damage.

The interplaying events occurring upon IR mainly involve oxidative stress. In fact, the dysfunctional mitochondria of cells under hypoxia show impaired electron flow and an enhanced formation and release of superoxide anion (O_2_^•−^) [15]. Moreover, at low O_2_ concentrations, the mitochondrial respiratory chain also generates nitric oxide (NO^•^) by nitrite reduction [16]. The O_2_^•−^ and NO^•^ generated under hypoxia promptly react to peroxynitrite (ONOO^−^) [17], which is regarded as the major harmful oxidant in IR damage [18]. In vivo, these mitochondrial mechanisms of oxidative stress are paralleled by inflammatory cell recruitment secondary to endothelial injury [19], which results in the further enhancement of ONOO^−^ generation. Of note, ONOO^−^ is held responsible for most, if not all, of the detrimental effects of excess NO^•^ [18,20].

The current study was designed to investigate whether the well-known protective action of RLX against IR-induced damage may be related to a beneficial redox balancing effect on target cells. To this purpose, we used a typical in vitro cellular model of hypoxia–reoxygenation (HR), equivalent to in vivo or ex vivo organ IR, on previously and successfully exploited H9c2 cardiac myoblasts, to demonstrate the cardioprotective role of RLX [13].

## 2. Materials and Methods

### 2.1. Cell Culture

H9c2 embryonic rat cardiac muscle cells, obtained from the European Collection of Cell Cultures (ECACC, Salisbury, UK), were cultured in Dulbecco’s modified Eagle’s medium (DMEM) supplemented with 10% heat-inactivated fetal bovine serum (FBS, Invitrogen, Carlsbad, CA, USA), 2 mmol L^−1^ glutamine, 250 U mL^−1^ penicillin G and 250 μg mL^−1^ streptomycin, in a humidified atmosphere with 5% CO_2_ at 37 °C. The H9c2 cells were subjected to hypoxia and reoxygenation (HR) as previously described [13]. Hypoxic challenge was achieved by using a modular incubator chamber (Billups-Rothenberg, Inc., San Diego, CA, USA) gassed with 95% N_2_ and 5% CO_2_. A flow meter was used to measure the quantity of gas mixture introduced into the chamber (25 L min^−1^). To simulate IR, after incubation under hypoxic conditions and in serum and glucose-free medium for 7 h, the cells were reoxygenated via the immediate replacement of fresh complete culture medium, and were returned to a normoxic environment at 37 °C for 60 min. Cell treatment was induced 24 h before the hypoxic challenge and at the moment of reoxygenation by adding RLX at concentrations of 17 nmol L^−1^ [15]. This RLX concentration was similar to that previously found to protect H9c2 cells from HR-induced injury [13]. The experiments were performed in triplicate.

### 2.2. Intracellular ROS and Mitochondrial O_2_^•−^

The ROS-sensitive fluorescent probe 2′,7′-dichlorodihydrofluorescein diacetate (H_2_DCFDA, 2.5 μmol L^−1^, Invitrogen, CA, USA) or the mitochondrial O_2_^•−^-specific fluorescent probe MitoSOX (3 μmol L^−1^, Invitrogen) dissolved in 0.1% DMSO and 0.01% *w/v* Pluronic acid F-127 were added to H9c2 cells seeded on glass coverslips for 15 min at 37 °C, as previously described [13]. Cells were fixed at room temperature for 10 min in 2% buffered paraformaldehyde and fluorescence analyzed using a Leica TCS SP8 confocal scanning microscope equipped with an argon laser source (excitation λ 488 nm or 543 nm, respectively) and a 63× oil immersion objective. ROS and mitochondrial O_2_^•−^ production were also estimated by flow cytometry [21]: briefly, single-cell suspensions were incubated with H_2_DCFDA (1 μmol L^−1^) or MitoSOX (0.5 μmol L^−1^) for 15 min at 37 °C, and immediately analyzed under a FACSCantoII flow cytometer (Becton–Dickinson) [22]. Data were analyzed using FACSDiva software (Becton Dickinson, San Jose, CA, USA).

### 2.3. Evaluation of Lipid Peroxidation

Lipid peroxidation, a reliable oxidative stress index, was assayed by confocal scanning microscopy using BODIPY 581/591 C11 (Life Technologies, Carlsbad, CA, USA), a lipophilic fluorescent probe that mimics the properties of natural lipids [13]. In the presence of oxidizing agents, BODIPY 581/591 C11 shifts its fluorescence from red to green. Cells, cultured on glass coverslips, were loaded with BODIPY dissolved in 0.1% DMSO (2.5 μmol L^−1^ final concentration) for 15 min at 37 °C in DMEM. Cells were then fixed in 2.0% buffered paraformaldehyde for 10 min at room temperature and fluorescence estimated using a confocal Leica TCS SP8 scanning microscope equipped with an argon laser source for fluorescence measurements. A series of optical sections (1024 × 1024 pixels) 1.0 μm in thickness was taken throughout the cell depth at intervals of 0.5 μm using a Leica Plan Apo 63× oil immersion objective and then projected as a single composite image by superimposition. Lipid peroxidation was also quantified by flow cytometry on single-cell suspensions incubated (30 min, 37 °C in the dark) with BODIPY 581/591 (2.5 μmol L^−1^) in DMEM, washed, resuspended in PBS and analyzed under a FACSCantoII flow cytometer (Becton Dickinson, San Jose, CA, USA).

### 2.4. Mitochondrial Activity

This was assayed by the resazurin reduction method (CellTiter-Blue, Promega Corp., ‎Madison, WI, USA), based on the ability of metabolically active cells to reduce resazurin to resorufin and dihydroresorufin proportionally to their number [23]. Conversion occurs intracellularly and is facilitated by mitochondrial, microsomal and cytosolic oxido-reductases. Resorufin produced by resazurin bioreduction was measured fluorometrically. Resazurin is not toxic to cells and maintains its stability in culture medium, allowing reliable measurements.

### 2.5. Mitochondrial Membrane Potential (Δψ)

Tetramethylrhodamine methyl ester perchlorate (TMRM), a lipophilic fluorescent dye, accumulates in mitochondria as a function of mitochondrial membrane potential Δψ [24]. For confocal microscopy, H9c2 cells were cultured on glass coverslips and loaded for 20 min at 37 °C with TMRM, and dissolved in 0.1% DMSO to a 100 nmol L^−1^ final concentration in the culture medium. The cells were fixed in 2% buffered paraformaldehyde for 10 min at room temperature, and the TMRM fluorescence was analyzed under a confocal Leica TCS SP8 scanning microscope equipped with a helium-neon laser source and a 63× oil immersion objective. Δψ was also quantified by flow cytometry on single-cell suspensions, washed twice with phosphate-buffered saline (PBS) and incubated for 20 min at 37 °C in the dark with TMRM dissolved in DMEM (100 nmol L^−1^). The cells were then rinsed, resuspended in PBS and analyzed under a FACSCantoII flow cytometer (Becton Dickinson).

### 2.6. Mitochondrial Transition Pore Opening (mPTP)

Mitochondrial permeability by transition pore opening is an index of mitochondrial dysfunction and early apoptosis. This was measured by the calcein fluorescence method [25], based on the principle that the fluorescent probe calcein-AM readily enters into cells and emits fluorescence upon de-esterification. The co-loading of cells with cobalt chloride, which cannot cross the mitochondrial membranes in living cells, quenches calcein fluorescence in the whole cell except mitochondria. During the induction of mPTP, cobalt can enter mitochondria and quenches calcein fluorescence, the decrease in which can be taken as a measure of the extent of mPTP induction. H9c2 cells grown on glass coverslips were loaded with calcein-AM (3 µmol L^−1^) and cobalt chloride (1 m mol L^−1^) for 20 min at 37 °C in DMEM. Following washing with PBS and fixation in 2% buffered paraformaldehyde for 10 min at room temperature, the cells were analyzed under a Leica TCS SP8 confocal laser scanning microscope equipped with an argon laser source (excitation λ 488 nm) and a 63× oil immersion objective. mPTP was also estimated by flow cytometry on single-cell suspensions incubated with calcein-AM (3 µmol L^−1^) and cobalt chloride (1 mmol L^−1^) for 20 min at 37 °C, washed twice with PBS and analyzed under a FACSCantoII flow cytometer (Becton Dickinson).

### 2.7. Caspase Activity

Since mitochondrial dysfunction triggers the apoptotic pathway, we next investigated the activation of pro-apoptotic initiator caspases 8 (extrinsic pathway) and 9 (intrinsic pathway), and effector caspase 3, as described [13]. H9c2 cells seeded on glass coverslips were incubated with a FAM-FLICA™ Caspase assay kit (Immunochemistry Technologies, Bloomington, MN, USA) for 30 min, following the manufacturer’s instructions. The FLICA reagent enters each cell and irreversibly binds to active caspases. Any unbound FLICA reagent diffuses out of the cell and is washed away. The remaining intracellular fluorescent signal is a direct measure of the active caspase enzyme activity present in the cell. After incubation, the cells were thoroughly washed and fixed in 2% buffered paraformaldehyde for 10 min at room temperature. Fluorescence was detected by a confocal Leica TCS SP8 scanning microscope equipped with an argon laser source and a 63× oil immersion objective. Caspase activity was also quantified by flow cytometry on single-cell suspensions treated with FAM-FLICA™ for 30 min at 37 °C, washed twice with PBS and estimated under a FACSCantoII flow cytometer (Becton Dickinson).

### 2.8. Cell Death Assay

Lactate dehydrogenase (LDH) activity, an index of cell death, was measured spectrophometrically in culture medium and in adherent H9c2 cells (to obtain total LDH content) using the LDH assay kit (Roche Diagnostics, Mannheim, Germany) as previously described [26]. LDH release was calculated as the percentage of total LDH content.

### 2.9. Superoxide Dismutase (SOD) Activity

Total SOD activity was measured using the Superoxide Dismutase Colorimetric Activity Kit (Invitrogen), according to the manufacturer’s protocol. Briefly, H9c2 cells from the different treatments were sonicated in cold PBS and the supernatant was used for the assay. The reaction was initiated by adding xanthine oxidase. SOD activity absorbance was measured at 450 nm on an Infinite M200PRO microplate reader (Tecan Group Ltd., Männedorf, Switzerland) and expressed as U/mg of total proteins. One unit of SOD is defined as the amount of enzyme causing half the maximum inhibition of 1.5 mmol L^−1^ blue tetrazolium reduction in the presence of riboflavin at pH 7.8 and 25 °C. Total protein content was measured spectrophotometrically using micro-BCA™ Protein Assay Kit (Pierce, IL, USA).

### 2.10. Glutathione Levels

To determine the level of intracellular GSH content, H9c2 single-cell suspensions were incubated in DMEM without serum and phenol red with the glutathione detection reagent ThiolTracker™ Violet (10 μmol L^−1^) (ThermoFisher Scientific, Waltham, MA USA), for 30 min at 37 °C, washed twice with PBS, and analyzed immediately by FACS.

### 2.11. Assay of RLX Antioxidant Properties

Finally, we assayed whether the RLX molecule was intrinsically endowed with chemical antioxidant properties. To this end, the ORAC (oxygen radical absorbance capacity) method was performed as previously described [27]. Briefly, fluorescein solution (6 nmol L^−1^) was freshly prepared daily in 75 mmol L^−1^ sodium phosphate buffer, pH 7.4, and Trolox (25–100 μmol L^−1^ final concentration) was used as a standard. To better reveal its possible antioxidant features, RLX was tested at a 100 μmol L^−1^ final concentration, which was substantially higher than that used in the other experiments. Seventy microliters of each sample with 100 μL of fluorescein was pre-incubated for 30 min at 37 °C in each well, before rapidly adding 2,2′-azobis(2-amidinopropane) dihydrochloride (AAPH) solution (19 mmol L^−1^ final concentration). Fluorescence was measured using a Synergy H1 microplate reader (BioTek, Winooski, VT, USA). The results were expressed as Trolox equivalents (nmol L^−1^) and then normalized for protein concentration.

### 2.12. Statistical Analysis

Unless otherwise indicated, statistical analysis of the differences between the quantitative values of the experimental groups was performed by one-way ANOVA and Newman–Keuls post-hoc tests for multiple comparisons. *p* < 0.05 was taken as the significance threshold.

## 3. Results

### 3.1. RLX Protects from HR-Induced Cellular Oxidative Stress

**Figure 1:**
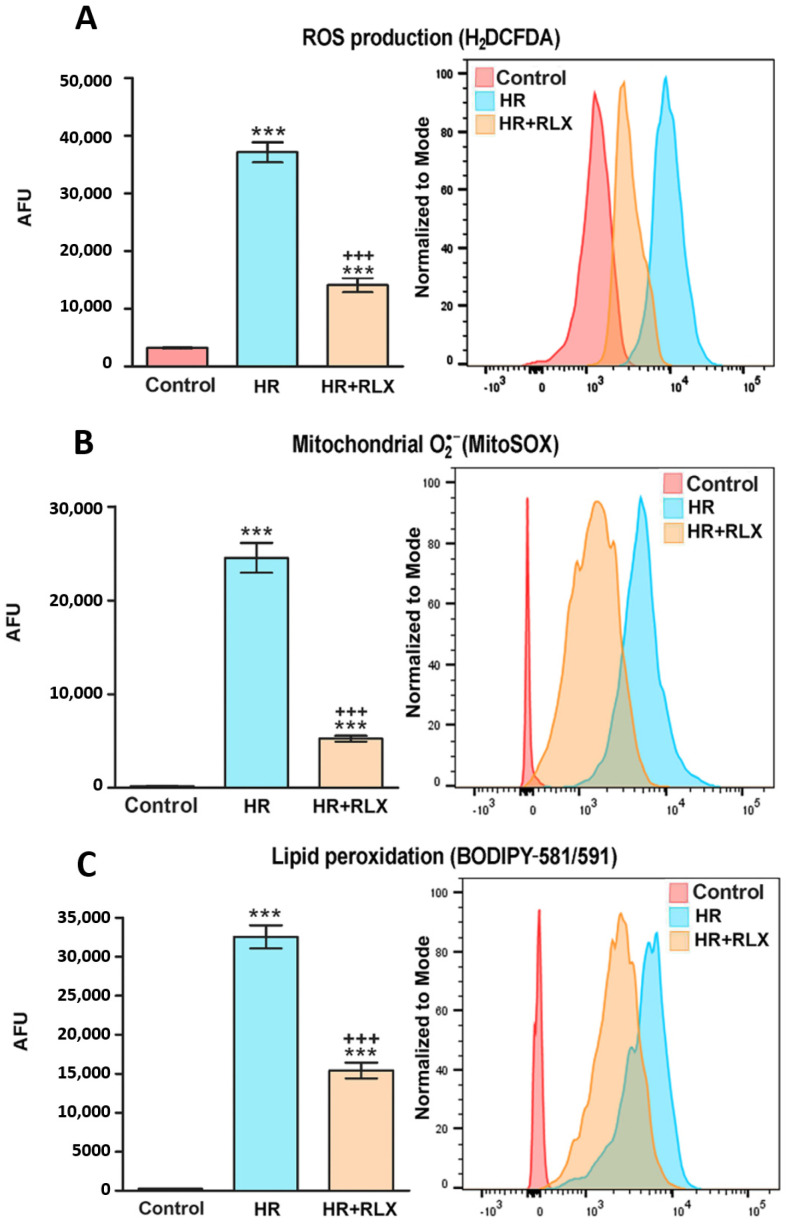
Quantitative evaluation and representative FACS diagrams of cellular oxidative stress assayed by fluorescent probes. (**A**) Endogenous ROS, determined by H_2_DCFDA; (**B**) mitochondrial O_2_^•−^ generation, determined by MitoSOX; (**C**) cell membrane lipid peroxidation, determined by BODIPY-581/591-C11. All these parameters of oxidative stress markedly increased upon hypoxia–reoxygenation (HR), an effect prevented by RLX (17 nmol L^−1^) added before hypoxia and at reoxygenation (AFU: arbitrary fluorescent units). Significance of differences (*n* = 3): *** *p* < 0.001 vs. controls, ^+++^ *p* < 0.001 vs. HR.

RLX (17 nmol L^−1^) added before hypoxia and at reoxygenation afforded protection to H9c2 cells by reducing the oxidative stress occurring upon HR, as previously reported [13]. Endogenously generated ROS, determined by loading the cells with the fluorescent probe H_2_DCFDA, was decreased more significantly in the RLX-treated cells than in the untreated control cells (Figure 1A). Similarly, the evaluation of mitochondrial O_2_^•−^ generation by the fluorescent probe MitoSOX showed that RLX significantly reduced O_2_^•−^-dependent fluorescence in comparison with the untreated controls (Figure 1B). We next assessed the peroxidation of cell membrane lipids, a typical detrimental effect of excess ROS, by the red-to-green fluorescence shift of BODIPY-581/591-C_11_ lysochrome. In keeping with the above findings, this oxidative stress parameter was increased by HR and significantly reduced by RLX (Figure 1C).

**Figure 2:**
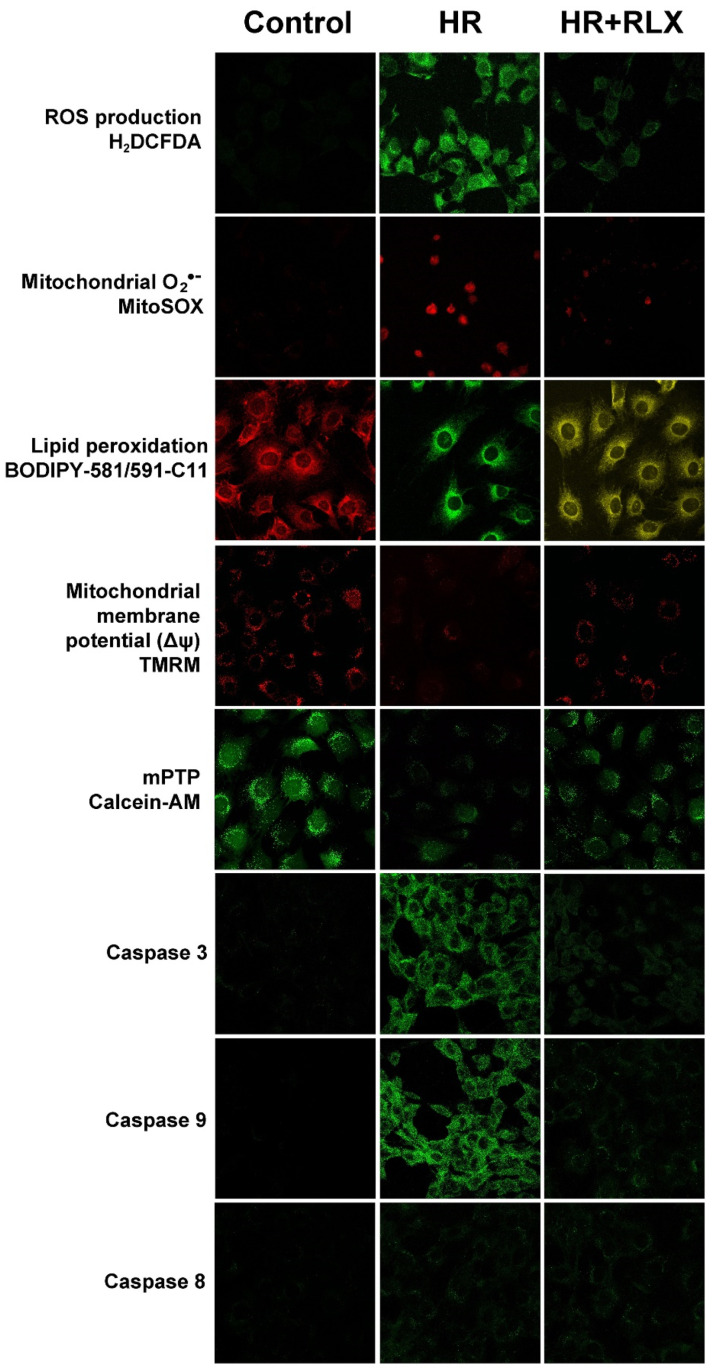
Representative confocal micrographs of H9c2 cells at the noted experimental conditions, examined after 1 h reoxygenation (HR, hypoxia–reoxygenation). Magnification, ×500.

Representative confocal images of these experiments performed after 1 h reoxygenation are shown in Figure 2.

### 3.2. RLX Improves Cell Mitochondrial Activity Impaired by HR

**Figure 3:**
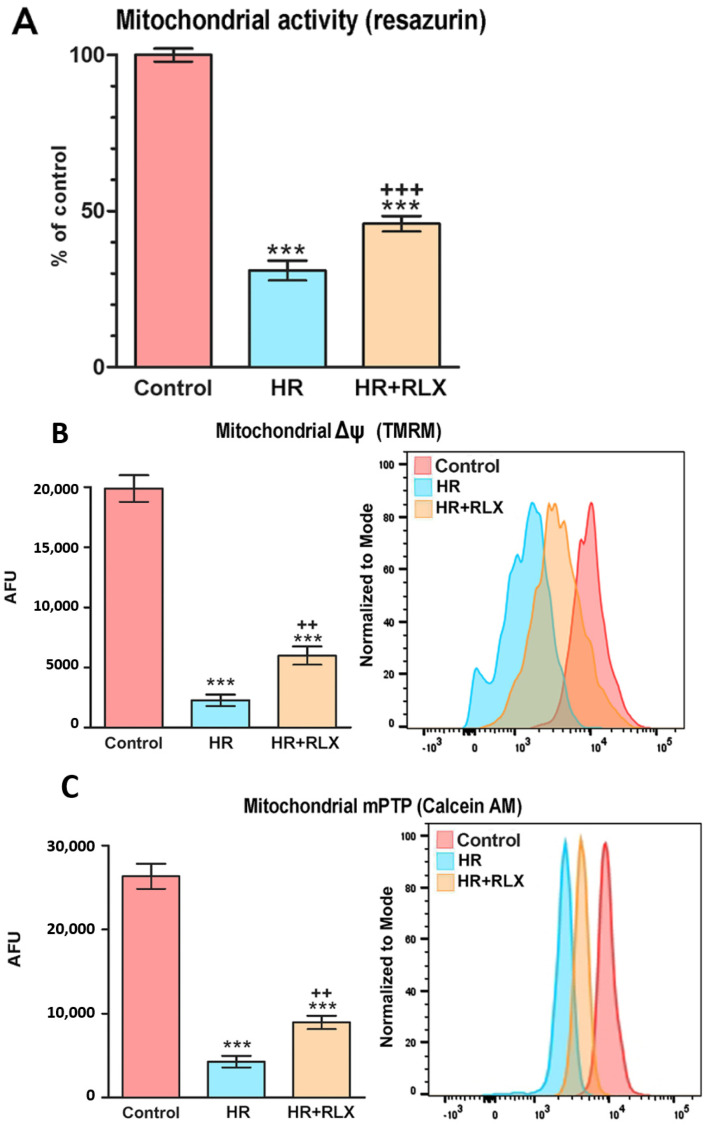
Quantitative evaluation and representative FACS diagrams of mitochondrial function assayed by fluorescent probes. (**A**) Efficiency of the respiratory chain, determined by reduced resazurin; (**B**) mitochondrial membrane potential (Δψ), determined by TMRM; (**C**) mitochondrial membrane transition pore opening (mPTP) determined by calcein extinction. All these parameters of mitochondrial functional impairment were markedly increased upon hypoxia–reoxygenation (HR), an effect prevented by RLX (17 nmol L^−1^) added before hypoxia and at reoxygenation. (AFU: arbitrary fluorescent units). Significance of differences (*n* = 3): *** *p* < 0.001 vs. controls, ^+++^ *p* < 0.001 ^++^ *p* < 0.01 vs. HR.

RLX (17 nmol L^−1^) added before hypoxia and at reoxygenation improved the assayed markers of the mitochondrial activity of H9c2 cells, which were markedly impaired by HR-induced oxidative stress. In fact, the efficiency of the mitochondrial respiratory chain, measured by the fluorescent signal of reduced resazurin (Figure 3A), and the mitochondrial membrane potential (Δψ), evaluated by the inlet of the fluorochrome TMRM (Figure 3B), were markedly reduced by HR and increased significantly in the RLX-treated cells. We next assayed the opening of transition pores in mitochondrial membranes (mPTP) by the extinction of calcein fluorescence (Figure 3C). This sensitive index of mitochondrial dysfunction and early apoptosis was increased by HR and significantly reduced by RLX.

Representative confocal images of these experiments performed after 1 h reoxygenation are shown in Figure 2.

### 3.3. RLX Protects from HR-Induced Cell Apoptosis

**Figure 4:**
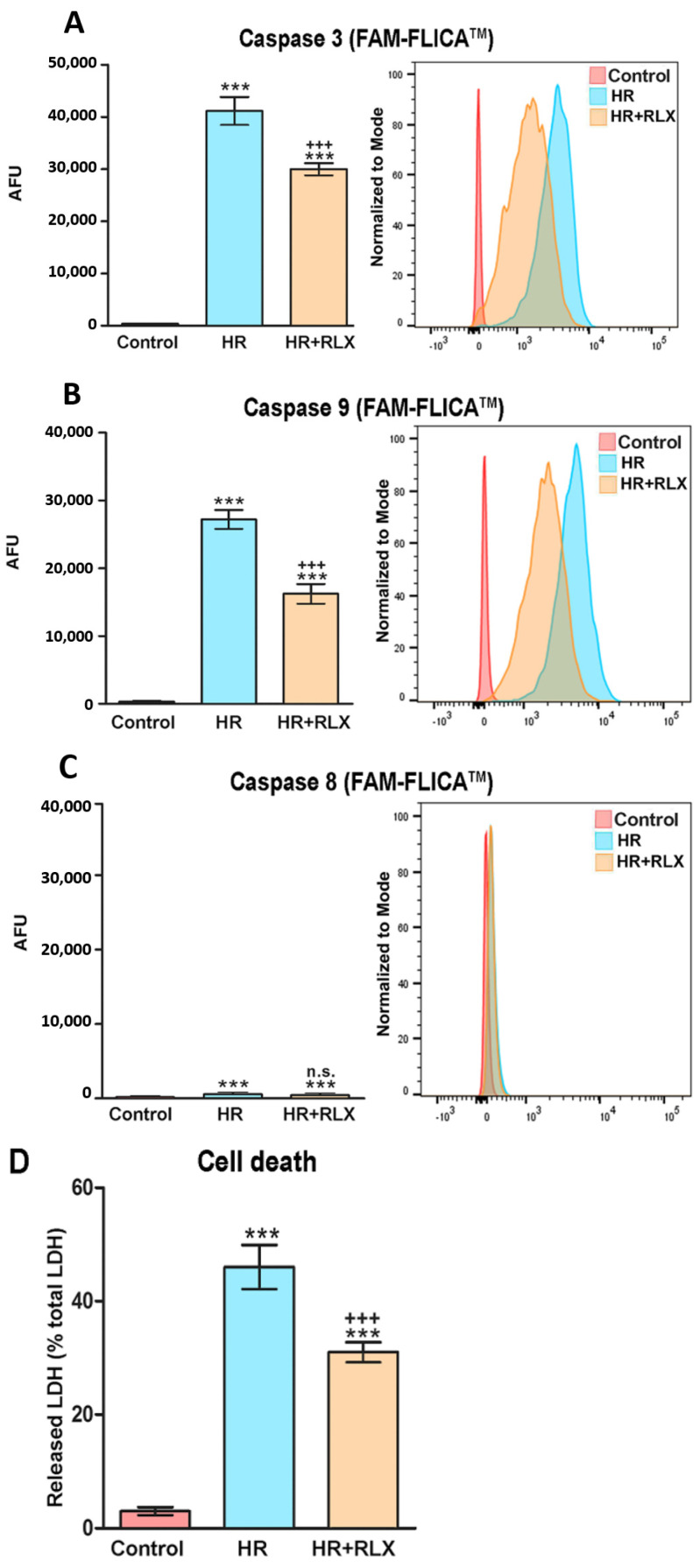
Quantitative evaluation and representative FACS diagrams of cellular apoptosis assayed by fluorescent probes. The activity of caspase 3 (**A**) and 9 (**B**), which are involved in the extrinsic apoptotic pathway, are markedly increased upon hypoxia–reoxygenation (HR), an effect prevented by RLX (17 nmol L^−1^) added before hypoxia and at reoxygenation. On the other hand, caspase 8 (**C**), which is involved in the intrinsic apoptotic pathway, is slightly (albeit significantly) increased by HR, but not substantially affected by RLX. Overall cell death (**D**), evaluated by LDH release, was also markedly increased by HR, and reduced by RLX. (AFU: arbitrary fluorescent units). Significance of differences (*n* = 3): *** *p* < 0.001 vs. controls, ^+++^ *p* < 0.001 vs. HR, n.s. not significant vs. HR.

Mitochondrial impairment is known to induce the intrinsic apoptotic pathway operated by the caspase 9/3 cascade [13]; therefore, we investigated if the protection afforded by RLX to H9c2 cells’ mitochondrial oxidative dysfunction, induced by HR, was accompanied by a reduction of apoptosis. Compared with the HR-exposed controls, the cells treated with RLX (17 nmol L^−1^) showed decreased activation of pro-apoptotic caspases 9 and 3, while caspase 8, involved in the extrinsic apoptotic pathway, was substantially unchanged (Figure 4A–C). Consistent with these findings, the evaluation of cell death by spectrophometric measurement of the LDH activity in the culture medium showed a marked rise in the HR-exposed controls, which was significantly reduced by RLX (Figure 4D).

Representative confocal images of these experiments performed after 1 h reoxygenation are shown in Figure 2.

### 3.4. Possible Mechanisms of the RLX’s Antioxidant Effects

**Figure 5:**
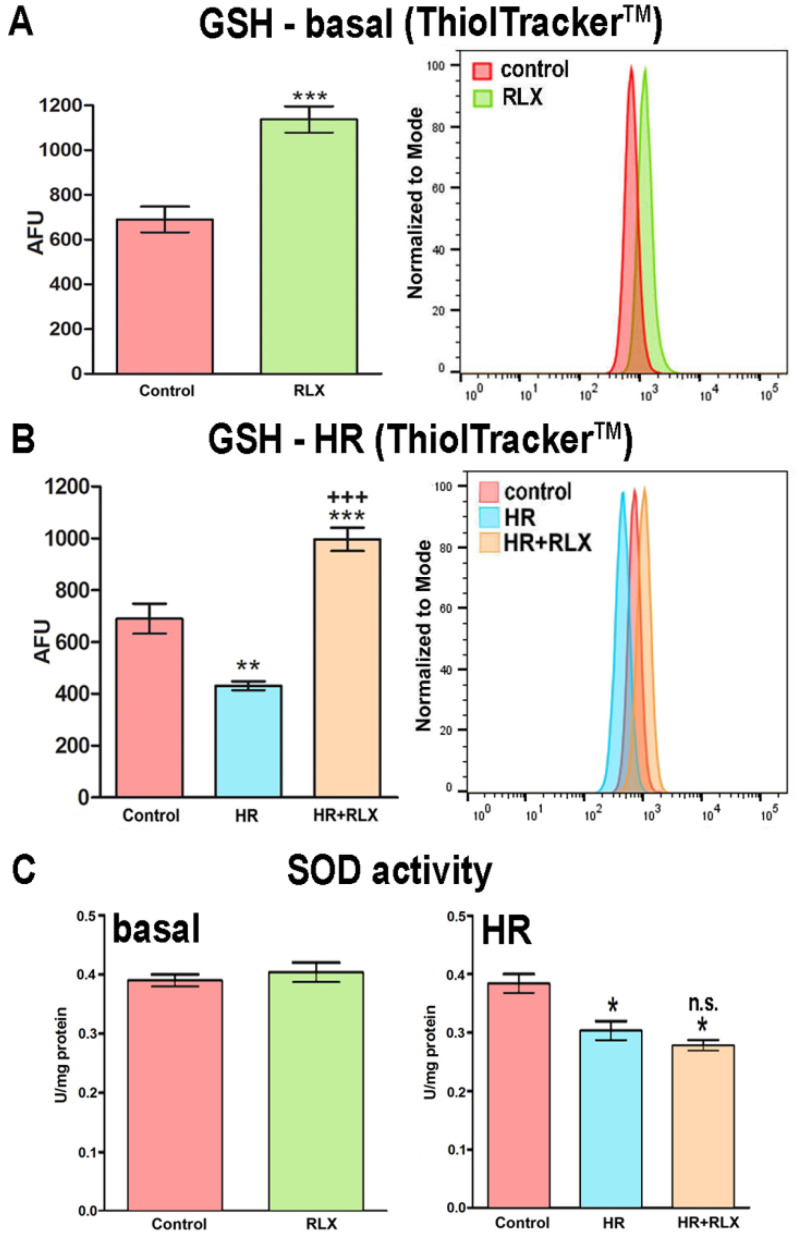
Quantitative evaluation and representative FACS diagrams of cellular antioxidant systems by fluorescent probes. Levels of reduced glutathione (GSH) in normal cells (**A**) and in cells subjected to HR (**B**), evaluated by ThiolTracker™ Violet. RLX markedly increases intracellular GSH in both normal and HR-exposed cells. Activity of superoxide dismutase (SOD) in normal cells and in cells subjected to HR (**C**). In either condition, RLX did not induce changes in SOD activity. (AFU: arbitrary fluorescent units). Significance of differences (*n* = 3): *** *p* < 0.001, ** *p* < 0.01, * *p* < 0.05 vs. controls; ^+++^ *p* < 0.001, n.s. not significant vs. HR.

**Figure 6:**
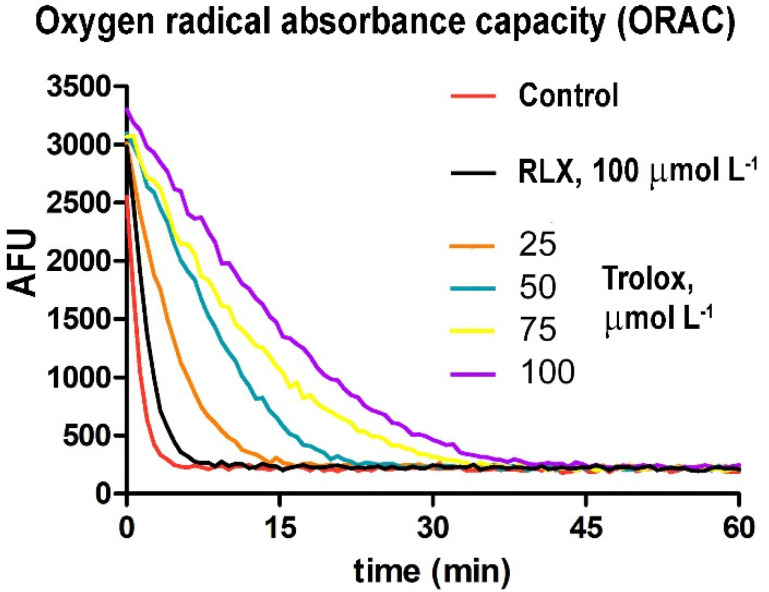
Assay of the chemical antioxidant properties of RLX. The concentration–response curve of O_2_^•−^ abatement in the presence of RLX showed no substantial antioxidant effects, at variance with increasing concentrations of the known antioxidant Trolox. Significance of differences (two-way ANOVA; *n* = 3): RLX vs. Trolox at any concentration, *p* < 0.001.

Finally, the possible mechanisms underlying the observed protection by RLX of HR-induced oxidative stress in H9c2 cells were investigated, starting from the reasoning that they may consist of (i) the induction of intracellular antioxidant pathways, and/or (ii) the direct antioxidant chemical properties of the RLX molecule. We first explored whether RLX was able to up-regulate two major antioxidant endogenous systems, i.e., reduced glutathione (GSH) and superoxide dismutase (SOD). The measurement of intracellular GSH levels in basal H9c2 cells not subjected to HR showed a marked, statistically significant increase in the RLX-treated cells as compared with the untreated controls (Figure 5A). Such RLX-induced GSH increase was even more prominent in the HR-exposed cells (Figure 5B). Conversely, the assay of SOD activity showed no significant differences between the RLX-treated and the untreated control cells, exposed or not to HR (Figure 5C). Compared with the controls, HR caused a slight (albeit significant) decrease in SOD activity (Figure 5C). We then evaluated by ORAC assay whether the RLX molecule exhibited specific chemical antioxidant activity, and we found that RLX (100 μmol L^−1^) displayed no direct antioxidant properties (Figure 6). The calculated antioxidant capacity of RLX was extremely low, being 0.77 nmol Trolox equivalents µg^−1^.

## 4. Discussion

An understanding of the key role of oxidative stress in the pathophysiology of IR is of pivotal importance in order to highlight the defensive mechanisms activated by cells and organs, as well as to identify effective therapeutic approaches [28]. Robust experimental evidence has been collected that RLX protects from IR-induced organ damage, not only because of indirect vasodilatory, anti-inflammatory and anti-fibrotic actions, but also via its direct cytoprotective effects, which increase the resistance of HR-exposed cells to oxygen deprivation and nitroxidative stress [4,5,6,7,8,9,10,11,12]. The current findings further strengthen and extend our previous reports that RLX blunts IR-induced injury and promotes the survival of H9c2 cardiac muscle cells subjected to HR [13]. They are also in keeping with the observation that RLX protects cardiomyocytes against oxidative stress-induced apoptosis [29]. In addition, the findings demonstrating that HR is associated with mitochondrial dysfunction, particularly increased O_2_^•−^ generation and decreased membrane potential (Δψ), support the current view that IR-induced cellular damage involves oxidative stress initiated by mitochondria [15]. The present findings on H9c2 cardiac muscle cells are in close agreement with those of previous studies on cultured neuroglial cells exposed to oxidative stress induced by oxygen and glucose starvation, which demonstrated that RLX afforded significant cytoprotection and the preservation of mitochondrial function [30]. The present study offers evidence that the cellular mechanisms underlying the protection afforded by RLX from HR-induced oxidative stress primarily involves an increase in the levels of reduced glutathione (GSH), a ubiquitous cellular antioxidant molecule. Of note, this effect of RLX had already manifested in control H9c2 cells not exposed to oxidative stress, suggesting that increased GSH bioavailability is a direct and specific action of RLX. This finding is in agreement with a previous report that RLX is able to increase GSH levels in H9c2 cells exposed to Cobalt chloride-induced oxidative damage [31]. The exact molecular mechanisms linking the activation of the RLX receptor and the up-regulation of GSH levels remain to be elucidated, and are an interesting matter for future investigations; anecdotal observations suggest the possible involvement of nuclear factor erythroid 2–related factor 2 (Nrf2), an emerging regulator of cellular resistance to oxidants [31]. On the other hand, in our experimental setup, RLX does not appear to involve the up-regulation of SOD activity, the key endogenous antioxidant enzymes that catalyze the dismutation and inactivation of O_2_^•−^ [32]. This is at variance with previous results obtained on H9c2 cells subjected to oxidative stress induced by exposure to high glucose [33], in which RLX appeared to increase MnSOD levels. Notably, in these experiments RLX was added to cells at very high concentrations (100 nmol mL^−1^), about 10,000 times higher than those we used in the present study. The present data also indicate that RLX does not possess intrinsic antioxidant properties. Of note, all cysteine residues—whose thiols can behave as reducing groups—that are present in the primary structure of both A and B RLX chains are involved in the formation of inter- and intra-chain disulfide bonds [34].

## 5. Conclusions

In conclusion, this study expands the current knowledge on the pharmacological properties of RLX as a molecule capable of reducing oxidative stress induced by conditions of oxygen deprivation followed by re-oxygenation (see graphical abstract). From a clinical perspective, this information joins the existing experimental background indicating RLX as a possible new drug for the primary and secondary prevention and therapy of ischemic diseases. Our study was performed using undifferentiated H9c2 myoblasts, raising questions concerning the relevance of the results obtained when compared to primary cardiomyocytes. However, in our previous works [13,26,35], we used a similar HR experimental approach using both H9c2 cardiomyoblasts and primary neonatal rat ventricular cardiomyocytes, and obtained comparable results. However, future in vivo experiments are needed to validate the present findings.
